# Mindfulness-Based Interventions for People with Autism Spectrum Disorder: A Systematic Literature Review

**DOI:** 10.3390/brainsci14101001

**Published:** 2024-09-30

**Authors:** Luca Simione, Alessandro Frolli, Francesca Sciattella, Salvatore Gaetano Chiarella

**Affiliations:** 1 Dipartimento di Scienze Umanistiche e Sociali Internazionali, Università degli Studi Internazionali, 00147 Rome, Italy; alessandro.frolli@unint.eu; 2 Istituto di Scienze e Tecnologie della Cognizione, Consiglio Nazionale delle Ricerche, 00185 Rome, Italy; 3 Scuola Internazionale Superiore di Studi Avanzati, 34136 Trieste, Italy

**Keywords:** mindfulness-based interventions, meditation, mental health, autism spectrum disorder, ASD

## Abstract

Background/Objectives: Mindfulness-based interventions (MBIs) have emerged in recent years as a strong candidate for the treatment of a range of difficulties faced by individuals with autism spectrum disorder (ASD), including cognitive, emotional, and social aspects. Therefore, we aimed to conduct a review that systematically examined the efficacy of MBIs for individuals with ASD and their caregivers. Methods: This systematic review was conducted according to the Preferred Reporting Items for Systematic Reviews and Meta-Analyses (PRISMA) guidelines. Our literature search was conducted within the MEDLINE database. We included in the review only longitudinal or intervention studies focusing mainly on mindfulness principles, while we excluded mixed intervention studies. We only included studies that explicitly utilized quantitative methodologies for evaluating the outcomes of the interventions, including mental health indices (e.g., stress, anxiety, depression) and assessments of cognitive and social skills (e.g., attention, prosociality). We conducted also a risk of bias assessment through the method of the Cochrane risk of bias tool for intervention studies ROBINS-I. Results: Thirty-seven studies were included in the review, and we grouped the studies by the targets of the interventions, i.e., adults (*n* = 12), children and adolescents (*n* = 9), caregivers and medical staff (*n* = 13), and combined intervention for both children/adolescents and their parents/caregivers (*n* = 5). The reviewed papers seem to support the feasibility and utility of mindfulness interventions for persons with ASD and their caregivers, but any recommendations based on this body of evidence should be made with caution due to the overall low quality of the studies conducted so far. Conclusions: The review reveals a positive outcome, including the alleviation of psychological distress, reduced behavioral problems, and enhanced cognitive and social skills in individuals with ASD. Despite such promising results, the review notes a limitation in the scarcity of MBIs for young patients, emphasizing caution in universally endorsing the existing literature. Moreover, the results underline the urgency of the exploration of tailored interventions for different ASD subgroups, considering varying levels of autism, and expanding support to teachers in educational settings.

## 1. Introduction

Mindfulness meditation, a family of body–mind techniques credited with enhancing psychological well-being across diverse populations [1,2], involves the cultivation of focused attention to the present-moment experience in a non-judgmental and non-reactive manner [3]. Central to mindfulness are two core skills: the awareness of present-moment experiences (i.e., the ‘what’ of mindfulness) and the fostering of acceptance and curiosity towards the content of these experiences (i.e., the ‘how’ of mindfulness) (e.g., [4,5]). The strength of mindfulness meditation lies in its inherent simplicity, facilitating acquisition within a brief timeframe of less than two months [6,7]. Empirical evidence demonstrates the effectiveness of mindfulness-based interventions (MBIs), i.e., interventions that exploited principles and techniques of mindfulness meditation, and in ameliorating various psychopathological symptoms, including stress, depression, anxiety, and aggressivity, among others (for recent reviews see, [8,9,10]. Moreover, meditation could also lead to an increase in dispositional mindfulness [11], which could lead to a further increase in mental health outcomes (e.g., [12]). Consequently, the practice of mindfulness meditation emerges as a promising avenue for the amelioration of psychological and neuropsychological symptoms in a diverse range of patients [12,13].

Focus attention meditation, often regarded as a foundational mindfulness exercise [14], involves the deliberate redirection of awareness to bodily sensations, such as the rhythmic movements associated with breathing or a detailed focus on specific body parts, as exemplified in breathing meditation and the body scan, respectively (for a review see [5,15]). These foundational practices, integral components of the widely recognized mindfulness-based stress reduction (MBSR) program [16], have been acknowledged for their efficacy in enhancing various cognitive and emotional dimensions. The benefits attributed to focus attention meditation encompass improvements in attentional capabilities, spanning executive functions [17], sustained attention [18,19], and heightened interoceptive awareness [20], all of which contribute to enhanced psychological well-being [6,21]. The accessibility of seated meditation with a focus on breathing sensations underscores its potential to augment individuals’ attentional resources while concurrently mitigating psychological distress (e.g., [22,23,24]). This underscores the significance of incorporating focus attention meditation into mindfulness interventions, highlighting its multifaceted impact on cognitive, emotional, and well-being domains [25,26]. Additionally, the empirical support provided by the MBSR program lends further credibility to the effectiveness of these practices in fostering a holistic improvement in individuals’ mental health [27].

Mindfulness appears to extend its positive influence also to social skills and prosocial behavior (see [28,29,30,31]). In this context, mindfulness is suggested to enhance awareness of others’ needs and emotions, fostering a deeper sense of connection with those around us [32,33,34]. Additionally, mindfulness is linked to an increase in compassion [35,36], defined as the ability to empathize with others’ suffering and take responsive action. Unlike empathy, which centers on recognizing others’ emotional experiences, compassion involves a proactive element, addressing others’ challenges and distress [37]. A mindfulness training program may significantly enhance social skills, including self- and other-oriented emotional awareness, coping mechanisms, emotional management skills, and a willingness to actively contribute to reducing others’ suffering while assisting them in achieving their needs [38,39]. Importantly, mindfulness is associated with a reduction in perceived social distance and isolation [22]. This effect is particularly noticeable in loving-kindness meditation, also known as ‘Metta’ meditation in Buddhist traditions [40]. This type of practice focuses on cultivating feelings of love, compassion, and goodwill towards oneself and others. During a loving-kindness session, practitioners extend positive wishes progressively to themselves, loved ones, friends, acquaintances, and even individuals with whom conflicts may arise, culminating in extending positive sentiments to all human and living beings. This practice is credited with fostering qualities such as self-care, gratitude, compassion, and prosocial behavior, and enhancing the ability to effectively manage challenging emotions (e.g., [41]), thereby significantly influencing socio-emotional processes. In summary, our review has pointed out several positive effects of mindfulness and MBIs on attention, awareness, and emotional processes. It is noteworthy that distinct benefits can be conveniently associated with different types of mindfulness practices, spanning from focused attention to the enriching practice of loving-kindness meditation [42,43]. Based on this premise, it seems that mindfulness interventions could be considered a strong candidate for the treatment of a range of psychological conditions.

Mindfulness interventions collected in the last fifty years have a vast amount of evidence supporting their usefulness for a variety of psychological and mental health disorders. In particular, the MBSR protocol was successful in reducing chronic stress, anxiety, and depression [44]. Moreover, it was applied successfully to patients with breast cancer [45], insomnia and sleep-related symptoms [46], and a range of chronic medical diseases [47]. Other interventions such as Mindfulness-Based Cognitive Therapy (MBCT) were designed explicitly to treat mood disorders and anxiety disorders [48], and it was proven effective in treating also a wide range of mental [49] and psychiatric disorders [50]. These MBIs aim to enhance an individual’s present-moment awareness and acceptance in a broad sense, and thus seem to be effective in targeting a range of mental health symptoms, with a neat beneficial effect. Mindfulness also positively affects cognitive functioning, such as attention and executive control [17,18], and social skills [29], both of which are often impaired in individuals with ASD. Thus, it holds potential applicability to autism spectrum disorder (ASD).

ASD is a complex condition marked by cognitive and emotional deficits [51]. On the cognitive side, ASD is associated with attentional and executive challenges, repetitive behaviors, impulse control issues, and difficulties in problem solving. On the emotional side, individuals with ASD often exhibit struggles in social interactions, communication, and emotional awareness and regulation. Given that mindfulness practices address both cognitive and emotional skills, it appears inherently well-suited as a therapeutic approach for individuals with ASD [52]. Nevertheless, recognizing the heterogeneity within the ASD spectrum is crucial, as symptoms, abilities, and impairment levels can widely vary among individuals [53,54]. ASD severity spans from mild to substantial problems, categorizing individuals into different levels of functioning [51]. High-functioning individuals possess relatively strong language and cognitive abilities while low-functioning individuals experience more significant impairments in communication, social interaction, and adaptive behavior. While mindfulness may effectively assist individuals with ASD in managing symptoms and cognitive deficits, its suitability may differ based on the individual’s level of functioning within the ASD spectrum [52].

Beyond individuals with ASD, another cohort that stands to benefit significantly from mindfulness exercises includes caregivers of ASD patients and healthcare staff working with them. These individuals often contend with heightened psychological distress and depression [55,56] as they navigate a range of emotional challenges in their daily interactions with ASD patients. Consequently, MBIs present a promising avenue for alleviating psychological distress and addressing emotional issues in those connected to individuals with ASD.

In the literature, many MBIs have been conducted for patients with ASD and their caregivers [57]. These interventions targeted different populations of patients, with different types of interventions and different outcomes. To better understand the actual literature landscape, we conducted a review of papers including an MBI for patients with ASD and persons related to them, such as caregivers and healthcare staff. We aimed to review such papers by categorizing them for the target population, type of interventions delivered, outcomes assessed, and actual effectiveness of the intervention, i.e., whether the intervention was effective in reducing symptoms or increasing psychological well-being and social or cognitive skills. In the existing literature, numerous MBIs have been implemented for both individuals with ASD and their caregivers [58]. These interventions have targeted diverse patient populations, employed varied intervention approaches, and assessed a range of outcomes. To provide a comprehensive overview of the current landscape, we conducted a review of the pertinent literature, specifically focusing on studies incorporating MBIs for individuals with ASD and their associated caregivers and healthcare staff. Our review aimed to categorize these studies based on the target population, intervention types, assessed outcomes, and the overall effectiveness of the intervention. This included an evaluation of whether the intervention demonstrated efficacy in symptom reduction, enhanced psychological well-being, and improved social and cognitive skills.

In contrast to prior reviews on this topic (e.g., see [57,58]), our current systematic literature review adopts a more focused approach. Specifically, we exclusively consider interventions that incorporate mindfulness-based exercises, while deliberately excluding any interventions of a ‘mixed’ nature. This exclusion encompasses approaches like Acceptance and Commitment Therapy (ACT; [59]) or other interventions that incorporate meditative techniques but are part of broader and more comprehensive therapeutic strategies. By refining our scope to mindfulness-based exercises alone, our review aims to provide a distinct and targeted analysis of the efficacy of these specific interventions for individuals with ASD and their associated caregivers and healthcare staff.

## 2. Materials and Methods

**Figure 1:**
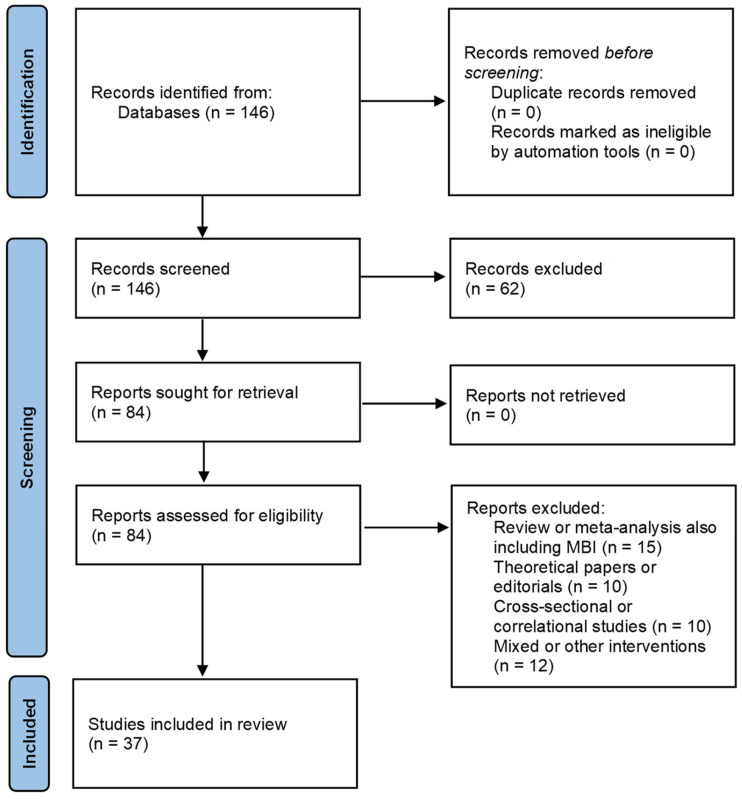
PRISMA flow diagram illustrating the selection process of studies for inclusion in this systematic review. The chart outlines the stages of identification, screening, eligibility, and inclusion, detailing the number of records identified through database searching, the number of records after duplicates were removed, the number of records screened, full-text articles assessed for eligibility, and the final number of studies included in the qualitative synthesis.

This systematic review was conducted according to the Preferred Reporting Items for Systematic Reviews and Meta-Analyses (PRISMA) guidelines, ensuring a transparent and replicable approach to the systematic review. The stages for the identification, screening, and inclusion of the studies that were found are shown in Figure 1.

### 2.1. Eligibility Criteria

In this systematic review, we focused on selecting manuscripts that implemented mindfulness-based interventions specifically for individuals with autism spectrum disorder (ASD), their caregivers, or healthcare professionals involved in their care. We excluded studies related to dispositional mindfulness, regardless of their design (cross-sectional or longitudinal), to maintain precision in our analysis. Only experimental studies incorporating a dedicated mindfulness intervention were considered for inclusion. Our primary interest lay in interventions emphasizing mindfulness practices, leading to the exclusion of studies that involved mixed interventions where mindfulness was only one of the included components, such as in ACT [59]. We included studies with a wide range of outcome measures, including mental health indices (e.g., stress, anxiety, depression) and assessments of cognitive and social skills (e.g., attention, prosociality). To ensure methodological rigor, we only included studies that explicitly utilized quantitative methodologies for evaluating the outcomes of the interventions.

### 2.2. Exclusion Criteria

We considered papers for review if they were written in English, empirical in nature, and had undergone peer review. Literature reviews and meta-analyses were excluded from our analysis. No restrictions were imposed based on participant demographics, allowing for the inclusion of studies irrespective of variables such as age, sex, socio-economic status, or the year of publication. This approach aimed to ensure a comprehensive examination of the available empirical evidence.

### 2.3. Search Strategy

Our literature search was conducted within the MEDLINE database via the PubMed interface, including papers published up to November 2023 (date of the database search). We used a unified search string employing Boolean operators ‘OR’ and ‘AND’, defined as follows: ((autism spectrum disorder) OR ASD OR Asperger) AND ((mindfulness OR meditation) AND (intervention)). This search string was applied to both the ‘Title/abstract’ and ‘MeSH terms’ fields. Specifically, the search terms were entered in the ‘Title’ field within the ‘Fields’ search box, and the ‘Date Published’ box was set to ‘All Years to Present’.

### 2.4. Study Selection

We made the selection process following the PRISMA guidelines (a full PRISMA 2020 Main Checklis can be retrieved in the Supplementary Materials). The search string produced a total of 146 papers. No duplicates were identified, so we proceeded to apply the inclusion and exclusion criteria. In total, 62 studies were excluded, two of which were not written in English and the others not related to either mindfulness interventions or ASD. After inspection of the title and abstract, we found that two non-English papers were also not relevant to our study. We then retrieved the remaining 84 papers to be analyzed. From this pool, we removed 10 papers describing cross-sectional or correlational studies, 10 papers including editorial or theoretical contributions about the usage of MBIs to treat ASD symptoms, and 15 papers were then excluded as they were literature reviews or meta-analyses also including MBIs but with a different focus in respect to the present study. Lastly, among the remaining 49 studies, we removed another 12 papers describing the results of studies based on mixed interventions, i.e., interventions including mindfulness practices or mindfulness-related techniques, among others, thus not mainly or solely based on mindfulness (e.g., ACT interventions). After applying the exclusion criteria, we selected a final pool of 37 papers including a mindfulness-based intervention for people with ASD or their caregivers. The studies included in the pool thus were classified as randomized controlled trials or as longitudinal studies.

### 2.5. Data Extraction

Data extraction was primarily handled by the first author (LS) and by the last author (SGC). They worked independently and then compared their results to a consensus. Before data extraction, the selection of the data to be extracted was conducted among all the authors. For each study, we decided to collect the following information: author(s), year of publication, number of participants enrolled, type of study, type of intervention, presence of a control group, main outcomes, and overall evaluation of result for the efficacy of the proposed intervention on the expected outcomes (as positive, neutral, or negative).

### 2.6. Risk of Bias Assessment

The overall quality of the studies was assessed by one investigator (LS) and then double-checked by other investigators (FS and SGC). To this aim, we utilized the Cochrane risk of bias tool for intervention studies ROBINS-I (Risk Of Bias In Non-randomized Studies of Interventions). We selected this tool over other tools specific for randomized trials as many of the studies retrieved did not include randomization or were pilot/feasibility studies with a single group. The ROBINS-I tool is designed to assess the risk of bias in non-randomized studies, such as observational studies or feasibility studies. It evaluates potential biases across seven key domains: confounding, selection of participants, classification of interventions, deviations from intended interventions, missing data, measurement of outcomes, and selection of reported results. The tool provides judgments on the level of risk in each domain, categorizing them as low risk, moderate risk, serious risk, or critical risk of bias, with an additional option for ‘no information’ when data are insufficient. This structured approach helps determine the overall validity and reliability of study findings. Irrespective of their quality, studies were kept in the systematic review. Table 1 reports a summary of bias evaluation, including author(s) and the year of publication of each article.

### 2.7. Classification and Theme Identification

The initial phase of the review involved screening titles and abstracts by one author (LS) using predefined search terms to ensure alignment with the eligibility criteria. Subsequently, a more in-depth analysis of the selected papers was conducted by another author (FS), who categorized them based on various dimensions: type of intervention, population or cohort under consideration, the primary focus of the papers (e.g., depression), and the study outcomes (significant or non-significant results). Each step of this process underwent a thorough review by all authors to achieve consensus on the proposed classifications. Following this, topics of investigation were collaboratively extracted by all authors and organized under overarching categories, as detailed in the subsequent section. This classification process aimed to provide an overview of the general landscape of the existing literature and to facilitate a focused exploration of predominant themes or topics emerging from the review. The collaborative approach ensured the reliability and comprehensiveness of the classification process. After scrutinizing the papers’ abstracts and full texts, we decided to group papers based on the target of the interventions included. Then, we obtained a final set of four groups of papers as follows: MBIs for adults with ASD (*n* = 12), MBIs for children and adolescents with ASD (*n* = 8), MBIs for caregivers and medical staff (*n* = 12), and combined intervention for both children/adolescents and their parents/caregivers (*n* = 5).

## 3. Results

### 3.1. Interventions for Adults with ASD

Adults with ASD have been the focus of researchers in this area. In total, we found twelve papers (see Table 2), nine of which broadly addressed adults with ASD, one focused on adult women [85], one on high-functioning adults [93], and one on adults with a co-occurrence of intellectual disability [72]. These studies investigated the effect of mindfulness interventions on depression, stress, anxiety, behavioral problems, emotion regulation, trait mindfulness, and quality of life among the autistic adult population. All yielded positive results utilizing MBI protocols and therapy, such as the widely used MBSR protocol in its original form (see [64,71,80,83]). We encountered only one instance of negative findings, which pertained to the study focused on a group of adult women with ASD [85]. On note, this study has been delivered online, while the others have been mainly conducted in presence. The effectiveness of the use of a meditation app to address depression showed instead favorable outcomes within the adult ASD population [94]. The study on adults with ASD and intellectual disability implemented the MindfulTEA program [72], an MBI specifically designed for those patients. Results revealed a significant decrease in self-injurious and aggressive/destructive behaviors after the intervention, but no significant changes were observed regarding stereotypical behavior. The effect of a modified MBT protocol (MBT-AS) was examined in a study involving high-functioning adults with ASD [93]. A significant reduction in depression, anxiety, and rumination was obtained in comparison with the control group.

### 3.2. Interventions for Children and Adolescents with ASD

The effect of MBI on the mental health and behavioral outcomes of children with ASD was evaluated in eight research papers (see Table 3). These studies scrutinized various variables including behavior, temper tantrums, adaptive coping strategies, social support seeking, executive functioning abilities, inhibition, selective attention, strengths and difficulties, social emotions, quality of life, and depressive symptoms. All these investigations yielded positive outcomes through MBIs, except for one that did not report significant differences in the outcomes assessed post-intervention [73]. Of note, two studies [67,68] adopted the TüTASS protocol, a structured group intervention focusing on self-perception and social skills tailored for children with ASD. These interventions highlighted the positive effects of incorporating mindfulness elements into structured interventions for this target population. Only four papers investigated instead adolescents with ASD, including outcomes of stress, anxiety, depression, and emotion regulation. One paper reported partial results [76], while the other three reported positive results [63,65,70]. The Emotional Awareness and Skills Enhancement individual therapy treatment [65], targeting emotion regulation impairments in adolescents with ASD, demonstrated feasibility, acceptability, and significant improvements in emotion regulation skills. Regarding anxious and depressive symptoms, a study was conducted with high-functioning patients, where mindfulness training (M-ERE) appeared more effective in managing anxiety and equally effective in the management of depressive symptoms compared with standard cognitive therapy [70].

### 3.3. Interventions for Caregivers of Individuals with ASD

Another substantial body of the literature concentrated on interventions targeting caregivers of individuals with ASD (see Table 4). This group comprised 12 papers, all including interventions for parental caregivers, with a sole exception involving professional staff working with ASD patients [90]. The interventions were evaluated based on various outcomes, including stress, anxiety, depression, life satisfaction, mindfulness, well-being, resilience, compassion, sleep, and emotional regulation. Many papers reported the outcomes of conventional mindfulness-based programs, such as the MBSR [69,81,84,87,88] or other generic mindfulness-based programs [61,90,91]. Additionally, other studies explored tailored interventions for caregivers, such as the Mindfulness Self-Care for Caregivers (MCSS; [95]), along with other types of MBIs [77,78]. Only one intervention was delivered online [79]. The findings predominantly indicated significant enhancements in stress reduction, emotional regulation, depression alleviation, and overall mental health improvement. Particularly noteworthy is the research involving professional staff [91], which provided a comparison across three experimental conditions: mindfulness training, psychoeducation, and in-service training-as-usual. In comparison to the other groups, the mindfulness-trained group exhibited a significant decrease in perceived stress, burnout, and symptoms of depression, coupled with an increase in compassion satisfaction.

### 3.4. Combined Interventions for Parents and Children/Adolescents with ASD

The final cluster of papers encompassed five mindfulness-based programs emerging as promising support for both young patients and their parents (see Table 5). Among these papers, three included adolescents with ASD as patients [66,74,86], while the other two focused on children with ASD [89,96]. These studies evaluated variables such as mindfulness, mental health, emotional regulation, autism symptoms, social communication problems, psychological stress, and cognitive and adaptive skills as outcomes. Overall, they reported positive results for all the assessed outcomes. The main intervention of this type, an interesting program implemented in four of the studies we evaluated, is the MYmind program [66,74,86,89], a mindfulness program in which youth with autism and their parents simultaneously receive group-specific mindfulness training. The MYmind program includes nine weekly 90 min mindfulness training sessions focused on various mindfulness techniques (e.g., meditations, breathing techniques, yoga poses). The program demonstrated the potential to contribute to social communication problems, emotional and behavioral regulation in youth with ASD, and mindfulness awareness, parenting, and social communication in parents. Notably, ref. [86] reported that the improvements shown by parents were still present at the 1-year follow-up.

### 3.5. Analsysis of Quality

Based on the risk of bias assessment of the 37 studies on mindfulness interventions for individuals with ASD, the overall quality of evidence could be considered medium. While solid and robust evidence collected through high-quality RCTs exists in the present literature [69,90], we included several underpowered, small studies. In general, adherence to the interventions was reported as high, as well as the adherence of therapists to the program. However, almost all studies relied on self-report questionnaires for evaluating their outcomes. Moreover, as participants in a therapeutic intervention such as an MBI could not be blind to the type of intervention itself, the likelihood that this factor altered the pattern of results should be considered at least as moderate for any of the reviewed studies. This is largely because blinding was often not feasible in these behavioral studies, leading to potential expectancy effects and performance bias. Another point of weakness in the existing literature is the inconsistency of interventions and outcomes evaluated across studies, which calls for caution while considering this emerging body of the literature as a whole in generalizing its result. We argue for future studies to converge on some promising interventions, such as the MYmind program for both parents and their children [66,74,86,89], or to opt for a well-structured program, such as the MBSR [16]. Lastly, while these studies provide some insights into the potential benefits of mindfulness practice for individuals with ASD, the evidence is not robust enough to support strong recommendations or large application of MBIs to individuals with ASD and their caregivers. Further high-quality research is necessary to provide more reliable and conclusive results in this area.

## 4. Discussion

In this review, we compiled evidence supporting the efficacy of mindfulness-based interventions (MBI) for individuals with autism spectrum disorder (ASD) and their caregivers. Given that mindfulness programs are recognized for enhancing cognitive skills [97,98] and prosocial behaviors [30] while reducing emotional distress [99] and dysregulation [100], they emerge as robust candidates for treating ASD symptoms. ASD core symptoms encompass impairments in both cognitive and social domains (e.g., [101]). To assess the feasibility and effectiveness of MBIs for individuals with ASD and their caregivers, we conducted a systematic search in the MEDLINE database, retrieving a total of 37 papers. These papers presented findings from various mindfulness interventions conducted with patients and caregivers. Overall, our research demonstrated the suitability and benefits of MBIs in this context, indicating a significant alleviation of psychological distress, decreased behavioral problems, and enhanced cognitive and social skills. Consequently, our results support the notion that MBIs present a promising avenue for intervention in patients with ASD, contributing to an overall reduction in ASD core symptoms.

### 4.1. Limitations and Strengths of the Reviewed Studies

The main limitation identified in our review is the scarce number of MBIs conducted on samples of young patients, with only seven papers presenting results from MBIs on groups of children or adolescents. Given this limited dataset, caution is warranted in providing a universally positive evaluation of the existing literature, although the results appear promising. Additionally, these papers highlighted various MBIs specifically designed for children with ASD, such as the TüTASS program [67,68] or tailored for school settings [75]. The utilization of a diverse type of mindfulness intervention further diminishes the possibility of drawing general conclusions about the overall effectiveness of these interventions. In this context, it becomes imperative to conduct replications of these interventions to validate their effectiveness on young patients with ASD. Moreover, standardizing protocols and programs through manualized approaches could enhance the ability to assess the effects of mindfulness on ASD symptoms more consistently and comparably. Addressing this issue in the current body of research underscores a critical imperative for future investigations in this domain.

On the other side, a higher number of intervention studies were dedicated to addressing adults with ASD symptoms, yielding a total of 12 identified studies. Notably, these studies employed manualized programs such as the MBSR program [3], providing a standardized framework for comparative analyses across various studies. This standardized approach facilitates comparisons not only within interventions targeting individuals with ASD but also between these interventions and those conducted on different patient cohorts or the general population. The results within this group of adult patients with ASD exhibited promise, particularly in reducing psychological distress. Interestingly, a singular study also documented the impact of mindfulness practices on ASD-related behavioral problems, specifically in self-injurious and aggressive/destructive behaviors [72]. It is noteworthy to highlight that this study involved an intervention specifically tailored for individuals with ASD and intellectual disabilities, emphasizing the feasibility of mindfulness programs even for low-functioning individuals. This is particularly significant as most mindfulness-based interventions for ASD have traditionally been proposed for high-functioning individuals (see [93]). Furthermore, ref. [72] demonstrated that interventions more attuned to the specific characteristics of individuals with ASD could be more effective in reducing ASD-related problems, while mindfulness programs designed for the general population exhibited efficacy primarily in reducing psychological distress [60,62,82,92]. This effect is consistent with the literature on mindfulness interventions for the general population, which mainly investigated the effect of MBIs on mental health [6,102,103].

In a similar vein, MBIs unsurprisingly demonstrated effectiveness in reducing psychological distress and mental health symptoms in caregivers of individuals with ASD. All 12 studies included in our review supported the feasibility and efficacy of MBIs for this population. This finding is consistent with previous systematic reviews that extensively documented the role of mindfulness programs in alleviating psychological problems in individuals (see [104]). For instance, meditation has proven beneficial not only in enhancing mental and physical health in patients with cancer [105] but also in their partners [106], who often experience stress in such challenging circumstances. Consequently, proposing an MBI for both patients and their caregivers could provide a familywise holistic treatment aimed at enhancing well-being at a broader level compared to interventions solely targeting patients.

### 4.2. Perspectives and Future Directions

In this context, an additional avenue for both clinical intervention and research could involve the development of combined interventions, where patients and caregivers undergo treatment either in separate groups or together in the same group. Several studies have provided interesting evidence of the feasibility of such combined interventions. Intervening with children or adolescents and their caregivers simultaneously has demonstrated a positive impact on both parties. The MYmind program [66] is a well-structured mindfulness-based nine-session parent and child training program that proposes a group-specific mindfulness approach. Due to its structured nature, this program has served as the framework for various studies [74,86,89], showcasing its effectiveness across different groups of patients. Furthermore, the program has been tested for children with ADHD and their parents [107,108], demonstrating the promising effectiveness of mindfulness as a feasible intervention for treating a range of neuropsychological disorders. Overall, based on the studies assessed in this paper, the combined interventions appear to hold the most promise. A potential avenue for future research could explore interventions with mixed groups, incorporating both parents and young individuals with ASD. Meditating together in the same group may cultivate a shared sense of mindfulness, contributing to improved overall well-being, adaptive functioning, increased social skills, and a reduction in loneliness for both parents and their children.

Based on our extensive review of the existing literature in the field, several future lines for research can be imagined. First, taking into consideration the predominant focus on adults with ASD in the studies evaluated in this paper, future research should explore tailored mindfulness-based interventions within specific subgroups, particularly children and adolescents. This extension of research could shed light on age-specific considerations and effectiveness, fostering a more comprehensive understanding of how mindfulness interventions can be optimized across different developmental stages. An additional avenue for future research lies in investigating the tailored needs of individuals with varying levels of autism and functioning. Understanding the issues for designing interventions that consider the specific challenges and strengths associated with different levels of ASD can contribute significantly to the refinement of mindfulness-based approaches for these people. Given that most combined interventions have identified parents as caregivers, there exists an opportunity to broaden the scope of research to include support for teachers in educational settings. Exploring the feasibility and impact of combined mindfulness protocols involving both parents and support teachers could offer valuable insights into extending the reach of mindfulness interventions within the educational context. Furthermore, future research could delve into determining the optimal duration and frequency of mindfulness interventions. Examining the long-term effects and conducting follow-ups in the studies can provide valuable information regarding the sustained efficacy of these interventions over time. This approach can contribute to the development of evidence-based recommendations for the implementation of mindfulness-based interventions, ensuring that they are both effective and feasible in various contexts.

### 4.3. Limitations

This study is not free from limitations. First, we only focused on intervention studies in which mindfulness was the main active component, while we discarded a quote from the relevant literature on ACT or mixed interventions which could be beneficial as well for individuals with ASD. Second, we only focused on the white literature published through the main scientific journal, leaving out all the gray literature from our analysis. While increasing the quality of the studies considered, this could potentially undermine or mask other effective capabilities of mindfulness in the treatment of ASD. Third, our study did not include a meta-analysis of the intervention outcomes. While such an analysis could have statistically supported the narrative findings of the positive effects of MBIs on ASD symptoms, the wide variety of protocols, study designs, and diverse outcomes across the studies reviewed made it challenging to conduct a robust and meaningful meta-analysis.

### 4.4. Conclusions

In conclusion, our review provides compelling evidence supporting the effectiveness of MBIs for individuals with ASD and their caregivers. These interventions have demonstrated positive outcomes, including alleviation of psychological distress, reduced behavioral problems, and improved cognitive and social skills in individuals with ASD. Notably, MBIs tailored for specific populations, such as children or adults with ASD, have shown promise in addressing core symptoms and enhancing overall mental health. While our review highlights the promising outcomes of MBIs for individuals with ASD and their caregivers, future research should focus on addressing identified limitations and further exploring the potential of combined interventions. Standardized protocols, replication studies, and a nuanced understanding of interventions across different age groups are crucial for advancing the field and maximizing the benefits of mindfulness-based approaches for individuals with ASD and their families.

## Figures and Tables

**Table 1:** Risk of bias assessment through the ROBINS-I tool.

Year	Authors	Confounding	Selection of Participants	Classification of Interventions	Deviations from Intended Interventions	Missing Data	Measurement of Outcomes	Selection of Reported Result
2023	Agius et al. [60]	M	L	L	L	L	M	L
2023	Bellone et al. [61]	M	M	L	NI	L	M	L
2022	Braden et al. [62]	M	L	L	L	M	L	L
2022	Clifford et al. [63]	M	L	L	L	L	L	L
2018	Conner and White [64]	S	M	L	NI	L	M	M
2019	Conner and White [65]	L	L	L	L	L	M	L
2014	De Bruin et al. [66]	M	L	L	L	M	M	L
2022	Drusedau et al. [67]	S	L	L	NI	L	M	M
2023	Drusedau et al. [68]	M	L	L	NI	L	M	M
2014	Dykens et al. [69]	M	L	L	L	L	L	L
2021	Frolli et al. [70]	M	L	L	NI	L	L	L
2020	Gaigg et al. [71]	S	L	L	L	M	M	L
2022	Gandia-Abellan et al. [72]	M	L	L	M	L	M	M
2023	Hatfield et al. [73]	M	M	L	NI	M	M	M
2021	Ho et al. [74]	M	L	L	L	L	L	L
2020	Juliano et al. [75]	M	M	L	NI	L	M	L
2022	Kemeny et al. [76]	S	L	L	M	L	M	L
2022	Ketcheson et al. [77]	S	M	L	L	M	M	M
2017	Lunsky et al. [78]	M	L	M	NI	L	M	L
2021	Lunsky et al. [79]	S	M	L	NI	M	M	L
2022	Lunsky et al. [80]	M	L	L	NI	L	M	L
2023	Neece et al. [81]	M	L	L	NI	L	M	L
2020	Pagni et al. [82]	M	L	L	NI	M	L	L
2023	Pagni et al. [83]	M	M	L	NI	M	L	L
2016	Rayan and Ahmad [84]	M	L	L	NI	M	M	S
2022	Redquest et al. [85]	S	M	L	NI	M	M	M
2018	Ridderinkhof et al. [86]	M	L	L	L	L	M	L
2021	Rojas-Torres et al. [87]	S	M	L	L	L	M	M
2023	Rojas-Torres et al. [88]	S	M	L	NI	L	M	M
2019	Salem-Guirgis et al. [89]	M	L	L	M	L	M	L
2020	Singh et al. [90]	M	L	L	L	L	L	L
2023	Singh et al. [91]	M	L	L	L	L	L	L
2017	Sizoo et al. [92]	M	L	L	L	M	M	L
2013	Spek et al. [93]	M	L	L	NI	L	M	L
2023	Stecher et al. [94]	M	M	L	L	L	L	L
2020	Weitlauf et al. [95]	L	L	L	L	L	M	M
2022	Weitlauf et al. [96]	M	L	L	L	M	M	M

Note. The risk of bias is graded as follows: L = low, M = moderate, S = serious, NI = No information is reported to adequately evaluate this domain.

**Table 2:** Mindfulness-based interventions for adults with ASD (*n* = 12).

Year	Authors	*n*	Type of Study	Control Group (If Any)	Intervention	Outcome	Result
2023	Stecher et al. [94]	125	RCT	Waitlist	8 weeks of daily meditation for 10 min with the Ten Percent Happier app.	Depression	Positive
2023	Agius et al. [60]	50	Intervention	None	8-week MBSR with reduced time of daily meditation (10–15 min).	Stress levels	Positive
2023	Pagni et al. [83]	96	RCT	Active	8-week MBSR with reduced group duration (2 h) and reduced practice time.	Depression, anxiety, mindfulness traits, autistic traits, and executive functioning abilities	Positive
2022	Braden et al. [62]	67	RCT	Active	8-week MBSR with reduced group duration (2 h) and no required full-day retreat.	Disability-related QoL	Positive
2022	Gandía-Abellán et al. [72]	15	Pilot	None	8-week MindfulTEA intervention, with 45 min weekly group session.	Behavioral problems	Positive
2022	Redquest et al. [85]	28	Pilot	None	6-week virtual MBI with 60 min weekly group session.	Psychological distress, self-compassion, mindfulness	Negative
2022	Lunsky et al. [80]	50	Intervention	None	6-week virtual MBI with 60 min weekly group session.	Mindfulness, self-compassion, distress	Positive
2020	Pagni et al. [82]	42	RCT	Active	8-week MBSR with reduced group duration (2 h) and reduced practice time.	Depression and anxiety	Positive
2020	Gaigg et al. [71]	54	RCT	Active	6–8 weeks of online courses including 10 exercises delivered through videos and audio files.	Anxiety	Positive
2018	Conner and White [64]	9	Pilot	None	6 weekly individual therapy sessions of 1 h based on MBCT, with reduced practice time.	Emotion regulation	Positive
2017	Sizoo and Kuiper [92]	59	RCT	Active	MBSR-inspired protocol with 13 weekly 90 min sessions.	Anxiety and depression	Positive
2013	Spek et al. [93]	42	RCT	Waitlist	MBT-AS protocol with 9 weekly group sessions plus 40–60 min of daily meditation.	Depression, anxiety, rumination	Positive

Note. RCT = Randomized controlled trial. MBSR = Mindfulness-based stress reduction. MBI = Mindfulness-based intervention.

**Table 3:** Mindfulness-based interventions for children and adolescents with ASD (N = 8).

Year	Authors	*n*	Type of Study	Control Group (If Any)	Intervention	Outcome	Result
2022 *	Drüsedau et al. [67]	25	Pilot	None	TüTASS program focused on mindful perception of body and emotions, delivered in 12 weekly 90 min sessions.	Depression	Positive
2023 *	Drüsedau et al. [68]	27	Intervention	None	TüTASS program delivered in 20 weekly 90 min sessions.	Stress levels	Positive
2023 *	Hatfield et al. [73]	14	Pilot	None	Mindful Movers program including 10 weekly 75 min sessions focused on mindful movement and breathing meditation.	Depression, anxiety, mindfulness traits, autistic traits, and executive functioning abilities	Negative
2022 *	Clifford et al. [63]	51	RCT	Active	Attention-based cognitive behavioral treatment based on MBCT and DBT delivered in 9 sessions of 60 min.	Disability-related QoL	Positive
2022	Kemeny et al. [76]	27	RCT	Waitlist	10 weeks of HeartMath Mindfulness protocol.	Behavioral problems	Positive
2021	Frolli et al. [70]	54	RCT	Active	Rational education training followed by 8 mindfulness-based sessions of 60 min.	Psychological distress, self-compassion, mindfulness	Positive
2020 *	Juliano et al. [75]	29	Pilot	None	Mindful Schools program delivered in 30 min sessions occurring twice per week for 8 weeks, focused on mindful breathing.	Mindfulness, self-compassion, distress	Positive
2019	Conner et al. [65]	20	Intervention	None	Emotional Awareness and Skills Enhancement (EASE), an individual therapy format delivered in 16 weekly 45–50 min sessions.	Anxiety	Positive

Note. RCT = Randomized controlled trial. Papers marked with an asterisk (*) included MBIs for children, while unmarked papers included MBIs for adolescents.

**Table 4:** Mindfulness-based interventions for caregivers of patients with ASD (*n* = 12).

Year	Authors	*n*	Type of Study	Control Group (If Any)	Intervention	Outcome	Result
2023	Neece et al. [81]	117	RCT	Active	MBSR intervention including eight weekly 2 h group sessions, a 6 h meditation retreat, and 30–45 min of daily home practice.	Stress	Positive
2023	Rojas-Torres et al. [88]	14	RCT	Waitlist	Mindfulness Parenting (MP) program with eight weekly sessions of 90 min, plus 15–30 min of daily practice.	Anxiety, parental stress, autism severity level, mindfulness awareness	Positive
2023	Bellone et al. [61]	13	Pilot	None	Mindful Self-Care for Caregivers (MSCC), including eight weekly 1.5 h classes plus home practice.	Mindfulness skills, sense of competency, stress, depression	Positive
2022	Ketcheson et al. [77]	27	RCT	Passive	MYtime, a mindfulness yoga program with a 60 min weekly class for 12 weeks.	Stress, anxiety and depression	Positive
2021	Lunsky et al. [79]	39	Pilot	None	Virtual six-week MBI based on MBCT with group session and brief audio practice recordings (10–15 min).	Mental health	Positive
2021	Rojas-Torres et al. [87]	12	RCT	Waitlist	MBSR + Self-compassion delivered in eight weekly sessions of 90 min plus 15–30 min practice at home.	Depression, anxiety, satisfaction in life, mindful attention awareness	Positive
2023 *	Singh et al. [91]	216	RCT	Active	MBI with 3 days training and a 32-week program based on standard Buddhist meditation practices.	Caregiver and client’s quality of life	Positive
2020 *	Singh et al. [90]	216	RCT	Active	MBI with 3 days training and a 32-week program based on standard Buddhist meditation practices.	Perceived stress, compassion satisfaction, compassion fatigue, depression	Positive
2020	Weitlauf et al. [95]	61	RCT	Active	Adapted MBSR with six one-hour individual sessions.	Stress, depression, anxiety, life satisfaction	Positive
2017	Lunsky et al. [78]	57	RCT	Active	Six 2 h weekly sessions of mindfulness training.	Psychological distress	Positive
2016	Rayan and Ahmad [84]	120	RCT	Passive	Brief five-week MBI with both in-person sessions and homework.	Psychological health domain of QoL, social health domain of QoL, mindfulness, positive stress reappraisal	Positive
2014	Dykens et al. [69]	243	RCT	Active	Standard MBSR program.	Stress, depression, anxiety, sleep, well-being	Positive

Note. The paper marked with an asterisk (*) included an MBI for healthcare staff, while unmarked papers included MBIs for parents. MBSR = Mindfulness-based stress reduction. MBI = Mindfulness-based intervention. RCT = Randomized controlled trial.

**Table 5:** Combined mindfulness-based interventions for children/adolescents with ASD and their caregivers (*n* = 5).

Year	Authors	*n*	Type of Study	Control Group (If Any)	Intervention	Outcome	Result
2022 *	Weitlauf et al. [96]	63	RCT	Active	Adapted MBSR with six one-hour individual sessions.	Depression	Positive
2021	Ho et al. [74]	37	RCT	Waitlist	MYmind program (see text for details).	Stress levels	Positive
2019 *	Salem-Guirgis et al. [89]	26	Intervention	None	MYmind program (see text for details).	Depression, anxiety, mindfulness traits, autistic traits, and executive functioning abilities	Positive
2018	Ridderinkhof et al. [86]	45	Intervention	None	MYmind program (see text for details).	Disability related QoL	Positive
2015	de Bruin et al. [66]	23	Intervention	None	MYmind program (see text for details).	Behavioral problems	Positive

Note. Papers marked with an asterisk (*) included MBIs for children, while unmarked papers included MBIs for adolescents. MBSR = Mindfulness-based stress reduction. RCT = Randomized controlled trial.

## Supplementary Materials

The following supporting information can be downloaded at: https://www.mdpi.com/article/10.3390/brainsci14101001/s1, PRISMA 2020 Main Checklist. Ref. [109] is cited in Supplementary Materials.

## References

[1] Khoury B., Sharma M., Rush S.E., Fournier C. (2015). Mindfulness-Based Stress Reduction for Healthy Individuals: A Meta-Analysis. J. Psychosom. Res..

[2] Waldron E.M., Hong S., Moskowitz J.T., Burnett-Zeigler I. (2018). A Systematic Review of the Demographic Characteristics of Participants in US-Based Randomized Controlled Trials of Mindfulness-Based Interventions. Mindfulness.

[3] Kabat-Zinn J. (2013). Some Reflections on the Origins of MBSR, Skillful Means, and the Trouble with Maps. Mindfulness.

[4] Shapiro S.L., Carlson L.E., Astin J.A., Freedman B. (2006). Mechanisms of Mindfulness. J. Clin. Psychol..

[5] Tang Y.-Y., Hölzel B.K., Posner M.I. (2015). The Neuroscience of Mindfulness Meditation. Nat. Rev. Neurosci..

[6] Querstret D., Morison L., Dickinson S., Cropley M., John M. (2020). Mindfulness-Based Stress Reduction and Mindfulness-Based Cognitive Therapy for Psychological Health and Well-Being in Nonclinical Samples: A Systematic Review and Meta-Analysis. Int. J. Stress Manag..

[7] Shapiro S.L., Brown K.W., Thoresen C., Plante T.G. (2011). The Moderation of Mindfulness-Based Stress Reduction Effects by Trait Mindfulness: Results from a Randomized Controlled Trial. J. Clin. Psychol..

[8] Gillions A., Cheang R., Duarte R. (2019). The Effect of Mindfulness Practice on Aggression and Violence Levels in Adults: A Systematic Review. Aggress. Violent Behav..

[9] Maddock A., Blair C. (2023). How Do Mindfulness-Based Programmes Improve Anxiety, Depression, and Psychological Distress? A Systematic Review. Curr. Psychol..

[10] Rodrigues M.F., Nardi A.E., Levitan M. (2017). Mindfulness in Mood and Anxiety Disorders: A Review of the Literature. Trends Psychiatry Psychother..

[11] Quaglia J.T., Braun S.E., Freeman S.P., McDaniel M.A., Brown K.W. (2016). Meta-Analytic Evidence for Effects of Mindfulness Training on Dimensions of Self-Reported Dispositional Mindfulness. Psychol. Assess..

[12] Geurts D.E., Haegens N.M., Van Beek M.H., Schroevers M.J., Compen F.R., Speckens A.E. (2021). Putting Mindfulness-Based Cognitive Therapy to the Test in Routine Clinical Practice: A Transdiagnostic Panacea or a Disorder Specific Intervention?. J. Psychiatr. Res..

[13] Giommi F., Simione L., Saldarini F., Raffone A., Ricci M.C., Sica C. (2023). The (In) Flexible Self: Psychopathology, Mindfulness, and Neuroscience. Int. J. Clin. Health Psychol..

[14] Anālayo B. (2019). Meditation on the Breath: Mindfulness and Focused Attention. Mindfulness.

[15] Yoshida K., Naito M., Kakigi R., Hasegawa C., Minami Y., Nakata H. (2020). Focused Attention Meditation Training Modifies Neural Activity and Attention: Longitudinal EEG Data in Non-Meditators. Soc. Cogn. Affect. Neurosci..

[16] Kabat-Zinn J. (2003). Mindfulness-Based Stress Reduction (MBSR). Constr. Hum. Sci..

[17] Lao S.-A., Kissane D., Meadows G. (2016). Cognitive Effects of MBSR/MBCT: A Systematic Review of Neuropsychological Outcomes. Conscious. Cogn..

[18] Giannandrea A., Simione L., Saldarini F., Fabbri M., Raffone A., Nolfi S. (2019). Effects of the Mindfulness-Based Stress Reduction Program on Mind Wandering and Dispositional Mindfulness Facets. Mindfulness.

[19] Petranker R., Eastwood J.D. (2021). Sitting with It: An Investigation of the Relationship between Trait Mindfulness and Sustained Attention. Conscious. Cogn..

[20] Hanley A.W., Mehling W.E., Garland E.L. (2017). Holding the Body in Mind: Interoceptive Awareness, Dispositional Mindfulness, and Psychological Well-Being. J. Psychosom. Res..

[21] Nyklíček I., Kuijpers K.F. (2008). Effects of Mindfulness-Based Stress Reduction Intervention on Psychological Well-Being and Quality of Life: Is Increased Mindfulness Indeed the Mechanism?. Ann. Behav. Med..

[22] Lindsay E.K., Creswell J.D. (2017). Mechanisms of Mindfulness Training: Monitor and Acceptance Theory (MAT). Clin. Psychol. Rev..

[23] Simione L., Pieroni I. (2023). Sensory-Processing Sensitivity as a Confounder in the Positive Relationship between Mindful Awareness and Psychological Distress: A Theoretical Review. Psychol. Conscious. Theory Res. Pract..

[24] Simione L., Saldarini F. (2023). A Critical Review of the Monitor and Acceptance Theory of Mindfulness. Mindfulness.

[25] Arpaia P., Pinto C.A., Rizzo A., Diacono D., Masi S., Villani M.L. Mindfulness-Based Emotional Acceptance in Combination with Neurofeedback for Improving Emotion Self-Regulation: A Pilot Study. Proceedings of the 2022 IEEE International Conference on Metrology for Extended Reality, Artificial Intelligence and Neural Engineering (MetroXRAINE).

[26] Roemer L., Williston S.K., Rollins L.G. (2015). Mindfulness and Emotion Regulation. Curr. Opin. Psychol..

[27] Accoto A., Tomasetti L., Nardi A., Fabbri M., Simione L. (2021). Beneficial Effects of Mindfulness-Based Stress Reduction Training on the Well-Being of a Female Sample during the First Total Lockdown Due to COVID-19 Pandemic in Italy. Int. J. Environ. Res. Public Health.

[28] Colombo S.L., Chiarella S.G., Raffone A., Simione L. (2023). Understanding the Environmental Attitude-Behaviour Gap: The Moderating Role of Dispositional Mindfulness. Sustainability.

[29] Hafenbrack A.C., Cameron L.D., Spreitzer G.M., Zhang C., Noval L.J., Shaffakat S. (2020). Helping People by Being in the Present: Mindfulness Increases Prosocial Behavior. Organ. Behav. Hum. Decis. Process..

[30] Luberto C.M., Shinday N., Song R., Philpotts L.L., Park E.R., Fricchione G.L., Yeh G.Y. (2018). A Systematic Review and Meta-Analysis of the Effects of Meditation on Empathy, Compassion, and Prosocial Behaviors. Mindfulness.

[31] Schindler S., Friese M. (2022). The Relation of Mindfulness and Prosocial Behavior: What Do We (Not) Know?. Curr. Opin. Psychol..

[32] Chiarella S.G., Colombo S.L., Raffone A., Simione L. (2020). Mindfulness Meditation Weakens Attachment to Self: Evidence from a Self vs. Other Binding Task. Mindfulness.

[33] Logie K., Frewen P. (2015). Self/Other Referential Processing Following Mindfulness and Loving-Kindness Meditation. Mindfulness.

[34] Trautwein F.-M., Naranjo J.R., Schmidt S., Schmidt S., Walach H. (2014). Meditation Effects in the Social Domain: Self-Other Connectedness as a General Mechanism?. Meditation—Neuroscientific Approaches and Philosophical Implications.

[35] Hildebrandt L.K., McCall C., Singer T. (2017). Differential Effects of Attention-, Compassion-, and Socio-Cognitively Based Mental Practices on Self-Reports of Mindfulness and Compassion. Mindfulness.

[36] Golden H.L., Vosper J., Kingston J., Ellett L. (2021). The Impact of Mindfulness-Based Programmes on Self-Compassion in Nonclinical Populations: A Systematic Review and Meta-Analysis. Mindfulness.

[37] Condon P., Karremans J.C., Papies E.K. (2017). Mindfulness, Compassion, and Prosocial Behaviour. Mindfulness in Social Psychology.

[38] Atkins P.W.B. (2013). Empathy, Self-Other Differentiation, and Mindfulness Training. Organizing through Empathy.

[39] Neff K.D., Dahm K.A., Ostafin B.D., Robinson M.D., Meier B.P. (2015). Self-Compassion: What It Is, What It Does, and How It Relates to Mindfulness. Handbook of Mindfulness and Self-Regulation.

[40] Salzberg S. (2011). Mindfulness and Loving-Kindness. Contemp. Buddhism.

[41] Przyrembel M., Vrticka P., Engert V., Singer T. (2019). Loving-Kindness Meditation—A Queen of Hearts? A Physio-Phenomenological Investigation on the Variety of Experience. J. Conscious. Stud..

[42] Nash J.D., Newberg A.B. (2023). An Updated Classification of Meditation Methods Using Principles of Taxonomy and Systematics. Front. Psychol..

[43] Yordanova J., Kolev V., Rothenberger A., Heinrich H. (2020). Common and Distinct Lateralised Patterns of Neural Coupling During Focused Attention, Open Monitoring and Loving Kindness Meditation. Sci. Rep..

[44] Li S.Y.H., Bressington D. (2019). The Effects of Mindfulness-based Stress Reduction on Depression, Anxiety, and Stress in Older Adults: A Systematic Review and Meta-analysis. Int. J. Ment. Health Nurs..

[45] Zhang Q., Zhao H., Zheng Y. (2019). Effectiveness of Mindfulness-Based Stress Reduction (MBSR) on Symptom Variables and Health-Related Quality of Life in Breast Cancer Patients—A Systematic Review and Meta-Analysis. Support. Care Cancer.

[46] Chen T.-L., Chang S.-C., Hsieh H.-F., Huang C.-Y., Chuang J.-H., Wang H.-H. (2020). Effects of Mindfulness-Based Stress Reduction on Sleep Quality and Mental Health for Insomnia Patients: A Meta-Analysis. J. Psychosom. Res..

[47] Bohlmeijer E., Prenger R., Taal E., Cuijpers P. (2010). The Effects of Mindfulness-Based Stress Reduction Therapy on Mental Health of Adults with a Chronic Medical Disease: A Meta-Analysis. J. Psychosom. Res..

[48] Goldberg S.B., Tucker R.P., Greene P.A., Davidson R.J., Kearney D.J., Simpson T.L. (2019). Mindfulness-Based Cognitive Therapy for the Treatment of Current Depressive Symptoms: A Meta-Analysis. Cogn. Behav. Ther..

[49] Galante J., Iribarren S.J., Pearce P.F. (2013). Effects of Mindfulness-Based Cognitive Therapy on Mental Disorders: A Systematic Review and Meta-Analysis of Randomised Controlled Trials. J. Res. Nurs..

[50] Chiesa A., Serretti A. (2011). Mindfulness Based Cognitive Therapy for Psychiatric Disorders: A Systematic Review and Meta-Analysis. Psychiatry Res..

[51] Lord C., Elsabbagh M., Baird G., Veenstra-Vanderweele J. (2018). Autism Spectrum Disorder. Lancet.

[52] Poquérusse J., Pagnini F., Langer E.J. (2021). Mindfulness for Autism. Adv. Neurodev. Disord..

[53] Lenroot R.K., Yeung P.K. (2013). Heterogeneity within Autism Spectrum Disorders: What Have We Learned from Neuroimaging Studies?. Front. Hum. Neurosci..

[54] Jeste S.S., Geschwind D.H. (2014). Disentangling the Heterogeneity of Autism Spectrum Disorder Through Genetic Findings. Nat. Rev. Neurol..

[55] Almansour M.A., Alateeq M.A., Alzahrani M.K., Algeffari M.A., Alhomaidan H.T. (2013). Depression and Anxiety among Parents and Caregivers of Autistic Spectrum Disorder Children. Neurosci. J..

[56] Alnazly E., Abojedi A. (2019). Psychological Distress and Perceived Burden in Caregivers of Persons with Autism Spectrum Disorder. Perspect. Psychiatr. Care.

[57] Hartley M., Dorstyn D., Due C. (2019). Mindfulness for Children and Adults with Autism Spectrum Disorder and Their Caregivers: A Meta-Analysis. J. Autism Dev. Disord..

[58] Cachia R.L., Anderson A., Moore D.W. (2016). Mindfulness in Individuals with Autism Spectrum Disorder: A Systematic Review and Narrative Analysis. Rev. J. Autism Dev. Disord..

[59] Hayes L., Boyd C.P., Sewell J. (2011). Acceptance and Commitment Therapy for the Treatment of Adolescent Depression: A Pilot Study in a Psychiatric Outpatient Setting. Mindfulness.

[60] Agius H., Bien A.M., Moniz J., Laird J., Whitehouse A.J.O., Chan W., Slattery M., Sprague K., Barone N., Suetani S. (2023). Mindfulness-Based Stress Reduction for Autistic Adults: A Feasibility Study in an Outpatient Context. Autism.

[61] Bellone K.M., Elliott S.C., Hynan L.S., Warren B., Jarrett R.B. (2023). Mindful Self-Care for Caregivers: A Proof of Concept Study Investigating a Model for Embedded Caregiver Support in a Pediatric Setting. J. Autism Dev. Disord..

[62] Braden B.B., Thomeer M.L., Therianou A., Villegas M.A., Adams J.W., Fath S.A., Roessler S. (2022). Quality of Life in Adults with Autism Spectrum Disorder: Influence of Age, Sex, and a Controlled, Randomized Mindfulness-Based Stress Reduction Pilot Intervention. Qual. Life Res..

[63] Clifford P., Gevers C., Jonkman K.M., Boer F., Begeer S. (2022). The Effectiveness of an Attention-Based Intervention for School-Aged Autistic Children with Anger Regulating Problems: A Randomized Controlled Trial. Autism Res..

[64] Conner C.M., White S.W. (2018). Brief Report: Feasibility and Preliminary Efficacy of Individual Mindfulness Therapy for Adults with Autism Spectrum Disorder. J. Autism Dev. Disord..

[65] Conner C.M., White S.W., Beck K.B., Golt J., Smith I.C., Mazefsky C.A. (2019). Improving Emotion Regulation Ability in Autism: The Emotional Awareness and Skills Enhancement (EASE) Program. Autism.

[66] de Bruin E.I., Blom R., Smit F.M., van Steensel F.J., Bögels S.M. (2015). MYmind: Mindfulness Training for Youngsters with Autism Spectrum Disorders and Their Parents. Autism.

[67] Drüsedau L., Götz A., Kleine Büning L., Conzelmann A., Renner T.J., Barth G.M. (2022). A Structured Group Intervention (TüTASS) with Focus on Self-Perception and Mindfulness for Children with Autism Spectrum Disorder, ASD: A Pilot Study. Eur. Arch. Psychiatry Clin. Neurosci..

[68] Drüsedau L.L., Götz A., Kleine Büning L., Conzelmann A., Renner T.J., Barth G.M. (2023). Tübinger Training for Autism Spectrum Disorders (TüTASS): A Structured Group Intervention on Self-Perception and Social Skills of Children with Autism Spectrum Disorder (ASD). Eur. Arch. Psychiatry Clin. Neurosci..

[69] Dykens E.M., Fisher M.H., Taylor J.L., Lambert W., Miodrag N. (2014). Reducing Distress in Mothers of Children with Autism and Other Disabilities: A Randomized Trial. Pediatrics.

[70] Frolli A., Ricci M.C., Di Carmine F., Orefice A., Saviano E., Carotenuto M. (2021). Emotional Rational Education Training Associated with Mindfulness for Managing Anxiety within Adolescents Affected by High-Functioning Autism: A Descriptive Study. Behav. Sci..

[71] Gaigg S.B., Cornell A.S., Bird G. (2020). Self-Guided Mindfulness and Cognitive Behavioural Practices Reduce Anxiety in Autistic Adults: A Pilot 8-Month Waitlist-Controlled Trial of Widely Available Online Tools. Autism.

[72] Gandía-Abellán H., Nieto C., García-Rubio C. (2023). Mindfulness for Adults with Autism Spectrum Disorder and Intellectual Disability: A Pilot Study. J. Intellect. Disabil..

[73] Hatfield M.K., Ashcroft E., Maguire S., Kershaw L., Ciccarelli M. (2023). “Stop and Just Breathe for a Minute”: Perspectives of Children on the Autism Spectrum and Their Caregivers on a Mindfulness Group. J. Autism Dev. Disord..

[74] Ho R.Y.F., Yeung P.K., Miu A.C., Chan C.C.H., Zhang Z., Cheung W.Y., Chan W.W. (2021). Brief Report: Mindfulness Training for Chinese Adolescents with Autism Spectrum Disorder and Their Parents in Hong Kong. J. Autism Dev. Disord..

[75] Juliano A.C., Alexander A.O., DeLuca J., Genova H. (2020). Feasibility of a School-Based Mindfulness Program for Improving Inhibitory Skills in Children with Autism Spectrum Disorder. Res. Dev. Disabil..

[76] Kemeny B., Burk S., Hutchins D., Gramlich C. (2022). Therapeutic Riding or Mindfulness: Comparative Effectiveness of Two Recreational Therapy Interventions for Adolescents with Autism. J. Autism Dev. Disord..

[77] Ketcheson L.R., Wengrovius C.M., Staples K.L., Miodrag N. (2022). MYTime: A Mindfulness and Yoga Program to Promote Health Outcomes in Parents of Children with Autism Spectrum Disorder. Glob. Adv. Health Med..

[78] Lunsky Y., Hastings R.P., Weiss J.A., Palucka A.M., Hutton S., White K. (2017). Comparative Effects of Mindfulness and Support and Information Group Interventions for Parents of Adults with Autism Spectrum Disorder and Other Developmental Disabilities. J. Autism Dev. Disord..

[79] Lunsky Y., Tint A., Robinson S., Khodeir M., Reaume C. (2021). Group Virtual Mindfulness-Based Intervention for Parents of Autistic Adolescents and Adults. J. Autism Dev. Disord..

[80] Lunsky Y., Tint A., Robinson S., Khodeir M., Reaume C. (2022). Virtual Group–Based Mindfulness Intervention for Autistic Adults: A Feasibility Study. Mindfulness.

[81] Neece C.L., Fenning R.M., Morrell H.E., Benjamin L.R. (2023). Comparative Effects of Mindfulness-Based Stress Reduction and Psychoeducational Support on Parenting Stress in Families of Autistic Preschoolers. Autism.

[82] Pagni B.A., Karvounides D., Zikopoulos B., Long L., Waldman A.T., Nauphal M., Datta S., Vangel M., Williams Z.M., Brown E.N. (2020). The Neural Correlates of Mindfulness-Induced Depression Reduction in Adults with Autism Spectrum Disorder: A Pilot Study. J. Neurosci. Res..

[83] Pagni B.A., Karvounides D., Zikopoulos B., Long L., Waldman A.T., Nauphal M., Datta S., Vangel M., Williams Z.M., Brown E.N. (2023). Distinct and Shared Therapeutic Neural Mechanisms of Mindfulness-Based and Social Support Stress Reduction Groups in Adults with Autism Spectrum Disorder. J. Psychiatry Neurosci..

[84] Rayan A., Ahmad M. (2016). Effectiveness of Mindfulness-Based Interventions on Quality of Life and Positive Reappraisal Coping among Parents of Children with Autism Spectrum Disorder. Res. Dev. Disabil..

[85] Redquest B., Tint A., St. John L., Hutton S., Palmer P., Lunsky Y. (2022). Virtual Group-Based Mindfulness Program for Autistic Women: A Feasibility Study. Womens Health.

[86] Ridderinkhof A., de Bruin E.I., Blom R., Bögels S.M. (2018). Mindfulness-Based Program for Children with Autism Spectrum Disorder and Their Parents: Direct and Long-Term Improvements. Mindfulness.

[87] Rojas-Torres L.P., Alonso-Esteban Y., López-Ramón M.F., Alcantud-Marín F. (2021). Mindfulness-Based Stress Reduction (MBSR) and Self Compassion (SC) Training for Parents of Children with Autism Spectrum Disorders: A Pilot Trial in Community Services in Spain. Children.

[88] Rojas-Torres L.P., Alcantud-Marín F., Alonso-Esteban Y. (2023). Mindfulness Parenting and Childish Play: A Clinical Trial with Parents of Children with Autism Spectrum Disorders. Psicothema.

[89] Salem-Guirgis S., de Bruin E.I., Blom R., Smit F.M., van Steensel F.J., Bögels S.M. (2019). MYmind: A Concurrent Group-Based Mindfulness Intervention for Youth with Autism and Their Parents. Mindfulness.

[90] Singh N.N., Lancioni G.E., Medvedev O.N., Hwang Y.-S., Myers R.E., Townshend K. (2020). Using Mindfulness to Improve Quality of Life in Caregivers of Individuals with Intellectual Disabilities and Autism Spectrum Disorder. Int. J. Dev. Disabil..

[91] Singh N.N., Lancioni G.E., Hwang Y.-S., Myers R.E., Townshend K., Medvedev O.N. (2023). Using Mindfulness to Improve Quality of Life in Caregivers of Individuals with Intellectual Disabilities and Autism Spectrum Disorder: Agency Outcomes for Caregivers and Clients. Adv. Neurodev. Disord..

[92] Sizoo B.B., Kuiper E. (2017). Cognitive Behavioural Therapy and Mindfulness Based Stress Reduction May Be Equally Effective in Reducing Anxiety and Depression in Adults with Autism Spectrum Disorders. Res. Dev. Disabil..

[93] Spek A.A., van Ham N.C., Nyklíček I. (2013). Mindfulness-Based Therapy in Adults with an Autism Spectrum Disorder: A Randomized Controlled Trial. Res. Dev. Disabil..

[94] Stecher C., Pagni B.A., Zikopoulos B., Brown E.N., Zomorrodi R. (2023). App-Based Meditation Habits Maintain Reductions in Depression Symptoms among Autistic Adults. Autism.

[95] Weitlauf A.S., Vehorn A.C., Taylor J.L., Warren Z.E., Brooks W., Martin K.L., Elkin S.M., Widaman K.F., Wallace E., McPheeters M.L. (2022). A Longitudinal RCT of P-ESDM with and without Parental Mindfulness Based Stress Reduction: Impact on Child Outcomes. J. Autism Dev. Disord..

[96] Weitlauf A.S., Vehorn A.C., Taylor J.L., Warren Z.E., Brooks W., Martin K.L., Elkin S.M., Widaman K.F., Wallace E., McPheeters M.L. (2020). Mindfulness-Based Stress Reduction for Parents Implementing Early Intervention for Autism: An RCT. Pediatrics.

[97] Chiesa A., Calati R., Serretti A. (2011). Does Mindfulness Training Improve Cognitive Abilities? A Systematic Review of Neuropsychological Findings. Clin. Psychol. Rev..

[98] Dunning D.L., Griffiths K., Kuyken W., Crane C., Foulkes L., Parker J., Dalgleish T. (2022). Do Mindfulness-Based Programmes Improve the Cognitive Skills, Behaviour and Mental Health of Children and Adolescents? An Updated Meta-Analysis of Randomised Controlled Trials. BMJ Ment. Health.

[99] Baroni D., Nerini A., Matera C., Stefanile C. (2018). Mindfulness and Emotional Distress: The Mediating Role of Psychological Well-Being. Curr. Psychol..

[100] Prakash R.S. (2021). Mindfulness Meditation: Impact on Attentional Control and Emotion Dysregulation. Arch. Clin. Neuropsychol..

[101] Gaigg S.B. (2012). The Interplay Between Emotion and Cognition in Autism Spectrum Disorder: Implications for Developmental Theory. Front. Integr. Neurosci..

[102] Janssen M., Heerkens Y., Kuijer W., Van Der Heijden B., Engels J. (2018). Effects of Mindfulness-Based Stress Reduction on Employees’ Mental Health: A Systematic Review. PLoS ONE.

[103] Zainal N.Z., Booth S., Huppert F.A. (2013). The Efficacy of Mindfulness-Based Stress Reduction on Mental Health of Breast Cancer Patients: A Meta-Analysis. Psychooncology.

[104] Jiménez-Gómez L., Yela J.R., Crego A., Melero-Ventola A.R., Gómez-Martínez M.Á. (2022). Effectiveness of the Mindfulness-Based Stress Reduction (MBSR) vs. the Mindful Self-Compassion (MSC) Programs in Clinical and Health Psychologist Trainees. Mindfulness.

[105] Ledesma D., Kumano H. (2009). Mindfulness-Based Stress Reduction and Cancer: A Meta-Analysis. Psychooncology.

[106] Birnie K., Garland S.N., Carlson L.E. (2010). Psychological Benefits for Cancer Patients and Their Partners Participating in Mindfulness-Based Stress Reduction (MBSR). Psychooncology.

[107] Valero M., Cebolla A., Colomer C. (2022). Mindfulness Training for Children with ADHD and Their Parents: A Randomized Control Trial. J. Atten. Disord..

[108] Zhang D., Zhang C., Sun T., Ma X., Wang Y., Wang L., Yu H. (2017). Mindfulness-Based Intervention for Chinese Children with ADHD and Their Parents: A Pilot Mixed-Method Study. Mindfulness.

[109] Page M.J., McKenzie J.E., Bossuyt P.M., Boutron I., Hoffmann T.C., Mulrow C.D., Shamseer L., Tetzlaff J.M., Akl E.A., Brennan S.E. (2020). The PRISMA 2020 statement: an updated guideline for reporting systematic reviews. MetaArXiv.

